# 9α-Acet­oxy-1β,10α-ep­oxy­parthenolide

**DOI:** 10.1107/S1600536810047471

**Published:** 2010-11-20

**Authors:** Mohamed Moumou, Mohamed Akssira, Ahmed El Hakmaoui, Lahcen Elammari, Ahmed Benharref, Moha Berraho

**Affiliations:** a Laboratoire de Chimie Bioorganique et Analytique, URAC 22, BP 146, FSTM, Université Hassan II, Mohammedia-Casablanca 20810 Mohammedia, Morocco; b Laboratoire de Chimie du Solide Appliquée, Faculté des Sciences, Avenue Ibn Battouta B.P. 1014 Rabat, Morocco; c Laboratoire de Chimie des Substances Naturelles, URAC16, Faculté des Sciences Semlalia, BP 2390 Bd My Abdellah, 40000 Marrakech, Morocco

## Abstract

The title compound, C_17_H_22_O_6_, was semi-synthesized from 9-hy­droxy­arthenolide, which was isolated from the chloro­form extract of the aerial parts of *Anvillea radiata*. The mol­ecule contains fused five- and ten-membered rings: the five-membered lactone ring has a twisted conformation, whereas the ten-membered ring displays an approximate chair–chair conformation. The dihedral angle between the rings is 24.76 (9)°.

## Related literature

For the isolation of 9-hy­droxy­arthenolide, see: El Hassany *et al.* (2004 ▶); Abdel Sattar *et al.* (1996 ▶). For the reactivity of this sesquiterpene, see: Castaneda-Acosta *et al.* (1993 ▶); Neukirch *et al.* (2003 ▶). For its biological activity, see: Abdel Sattar *et al.* (1996 ▶). For ring puckering parameters, see: Cremer & Pople (1975 ▶). For conformations of ten-membered rings, see: Castaneda-Acosta *et al.* (1997 ▶); Watson & Zabel (1982 ▶); Moumou *et al.* (2010 ▶).
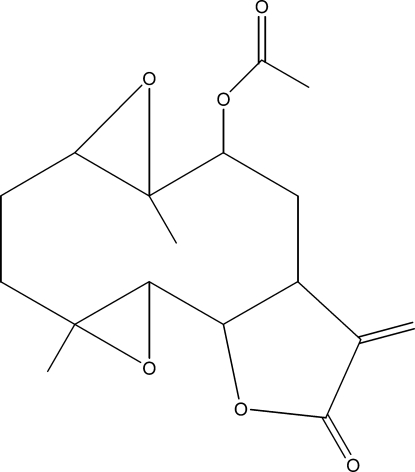


## Experimental

### 

#### Crystal data

C_17_H_22_O_6_
*M* *_r_* = 322.35
Monoclinic, 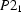

*a* = 8.2390 (3) Å
*b* = 10.6482 (4) Å
*c* = 9.4633 (3) Å
β = 102.039 (2)°
*V* = 811.96 (5) Å^3^
*Z* = 2
Mo *K*α radiation
μ = 0.10 mm^−1^
*T* = 298 K
0.38 × 0.27 × 0.12 mm

#### Data collection

Bruker X8 APEX CCD area-detector diffractometer
12911 measured reflections
2718 independent reflections
2480 reflections with *I* > 2σ(*I*)
*R*_int_ = 0.023

#### Refinement

*R*[*F* ^2^ > 2σ(*F* ^2^)] = 0.038
*wR*(*F* ^2^) = 0.115
*S* = 1.05
2718 reflections
211 parameters
1 restraint
H-atom parameters constrained
Δρ_max_ = 0.25 e Å^−3^
Δρ_min_ = −0.17 e Å^−3^

### 

Data collection: *APEX2* (Bruker, 2007 ▶); cell refinement: *SAINT* (Bruker, 2007 ▶); data reduction: *SAINT*; program(s) used to solve structure: *SHELXS97* (Sheldrick, 2008 ▶); program(s) used to refine structure: *SHELXL97* (Sheldrick, 2008 ▶); molecular graphics: *ORTEP-3 for Windows* (Farrugia, 1997 ▶); software used to prepare material for publication: *WinGX* (Farrugia, 1999 ▶).

## Supplementary Material

Crystal structure: contains datablocks I, global. DOI: 10.1107/S1600536810047471/fj2367sup1.cif

Structure factors: contains datablocks I. DOI: 10.1107/S1600536810047471/fj2367Isup2.hkl

Additional supplementary materials:  crystallographic information; 3D view; checkCIF report

## Figures and Tables

**Fig. 1.**
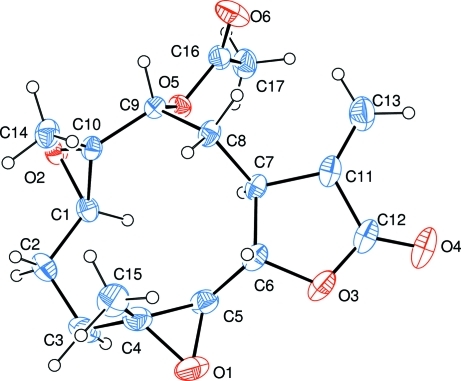
Molecular structure of the title compound with the atom-labelling scheme. Displacement ellipsoids are drawn at the 30% probability level. H atoms are represented as small spheres of arbitrary radii.

## supplementary crystallographic information

### Comment

The Natural sesquiterpene lactone (9α-hydroxyparthenolide) is the main constituent of the chloroform extract of aerial parts of Anvillea radiata (El Hassany *et al.*, 2004), and of Anvillea garcini(Abdel Sattar *et al.*,1996). The reactivity of this sesuiterpène and its derivatives has been the subject of several studies (Castaneda-Acosta *et al.*,1993; Neukirch *et al.*, 2003), in order to prepare products with high value added, used in industrial pharmacology. In the same context, we carried out the acetylation followed by epoxydation of 9α-hydroxyparthenolide. Thus, the action of one equivalent of acetic anhydride on this sesquiterpene in pyridine at 0°C leads to quantitative yield 9α-*ac*étoxyparthenolide. The treatment of this latter with one equivalent of *meta*-choroperbenzoïque acid (mCPBA) in dichloromethane at room temperature gives 9α-acetoxy-1β, 10β-epoxyparthenolide with a yield of 95%. The structure of this new product was determined by NMR spectral analysis of 1H, 13 C and mass spectrometry, and confirmed by a study of *X* ray crystallography. The structure of (I) was established by 1H and 13 C NMR and confirmed by its single-crystal X-ray structure.The molecule is built up from two fused five-and ten-membered rings.(Fig. 1). The five-membered ring adopts a twisted conformation,as indicated by Cremer & Pople (1975) puckering parameters Q = 0.26 (2) Å and φ = 23.77 (4)°. The ten-membered ring displays an approximate chair-chair conformation. This is the typical conformation observed for other sesquiterpenes lactones (Moumou *et al.*, 2010; Watson & Zabel, 1982; Castaneda-Acosta *et al.*, 1997).

### Experimental

To a solution of 1,2 g (4,54 mmol) of 9α-hydroxyparthenolide in 30 ml of pyridine was added 10 ml of acetic anhydride. The mixture is left stirring for 12 h at room temperature and then treated with 100 ml of ice water and extracted with chloroform. The residue obtained after drying and evaporation of solvent was chromatographed on silica gel eluting with hexane-ethyl acetate (80/20) and allowed to isolate in pure form with a yield qantitatif the 9α-*ac*étoxyparthenolide. To 0.5 g (1,6 mmol) of this latter dissolved in 40 ml of dichloromethane is added an equivalent of acid *meta* chloroperbenzoïque (mCPBA). The reaction mixture was stirred at room temperature for 3 h, then treated with a solution of sodium bisulfite at 10% and extracted with dichloromethane. The organic phase is dried over sodium sulfate and then evaporated under vacuum. chromatography of the residue obtained on silica gel column eluting with hexane ethyl acetate (75/25), allowed us to obtain the 9α-Acetoxy-1β, 10α-epoxyparthenolide with a yield of 80%.Crystallization of this product was carried out at room temperature from an ethyl acetate solution.

### Refinement

All H atoms were fixed geometrically and treated as riding with C—H = 0.96 Å (methyl),0.97 Å (methylene), 0.98Å (methine) with *U*_iso_(H) = 1.2Ueq(methylene, methine and OH) or *U*_iso_(H) = 1.5Ueq(methyl). In the absence of significant anomalous scattering, the absolute configuration could not be reliably determined and thus 2417 Friedel pairs were merged and any references to the Flack parameter were removed.

### Crystal data

**Table:**

C_17_H_22_O_6_	*F*(000) = 344
*M**_r_* = 322.35	*D*_x_ = 1.318 Mg m^−^^3^
Monoclinic, *P*2_1_	Mo *K*α radiation, λ = 0.71073 Å
Hall symbol: P 2yb	Cell parameters from 12911 reflections
*a* = 8.2390 (3) Å	θ = 2.2–31.0°
*b* = 10.6482 (4) Å	µ = 0.10 mm^−^^1^
*c* = 9.4633 (3) Å	*T* = 298 K
β = 102.039 (2)°	PRISM, colourless
*V* = 811.96 (5) Å^3^	0.38 × 0.27 × 0.12 mm
*Z* = 2	

### Data collection

**Table:**

Bruker X8 APEX CCD area-detector diffractometer	2480 reflections with *I* > 2σ(*I*)
Radiation source: fine-focus sealed tube	*R*_int_ = 0.023
graphite	θ_max_ = 31.1°, θ_min_ = 2.2°
φ and ω scans	*h* = −11→11
12911 measured reflections	*k* = −15→15
2718 independent reflections	*l* = −13→13

### Refinement

**Table:**

Refinement on *F*^2^	Primary atom site location: structure-invariant direct methods
Least-squares matrix: full	Secondary atom site location: difference Fourier map
*R*[*F*^2^ > 2σ(*F*^2^)] = 0.038	Hydrogen site location: inferred from neighbouring sites
*wR*(*F*^2^) = 0.115	H-atom parameters constrained
*S* = 1.05	*w* = 1/[σ^2^(*F*_o_^2^) + (0.0742*P*)^2^ + 0.0491*P*] where *P* = (*F*_o_^2^ + 2*F*_c_^2^)/3
2718 reflections	(Δ/σ)_max_ < 0.001
211 parameters	Δρ_max_ = 0.25 e Å^−^^3^
1 restraint	Δρ_min_ = −0.17 e Å^−^^3^

### Special details

**Table:**

Geometry. All e.s.d.'s (except the e.s.d. in the dihedral angle between two l.s. planes) are estimated using the full covariance matrix. The cell e.s.d.'s are taken into account individually in the estimation of e.s.d.'s in distances, angles and torsion angles; correlations between e.s.d.'s in cell parameters are only used when they are defined by crystal symmetry. An approximate (isotropic) treatment of cell e.s.d.'s is used for estimating e.s.d.'s involving l.s. planes.
Refinement. Refinement of *F*^2^ against ALL reflections. The weighted *R*-factor *wR* and goodness of fit *S* are based on *F*^2^, conventional *R*-factors *R* are based on *F*, with *F* set to zero for negative *F*^2^. The threshold expression of *F*^2^ > σ(*F*^2^) is used only for calculating *R*-factors(gt) *etc*. and is not relevant to the choice of reflections for refinement. *R*-factors based on *F*^2^ are statistically about twice as large as those based on *F*, and *R*- factors based on ALL data will be even larger.

### Fractional atomic coordinates and isotropic or equivalent isotropic displacement parameters (Å^2^)

**Table:**

	*x*	*y*	*z*	*U*_iso_*/*U*_eq_	
C14	0.6599 (2)	−0.1768 (2)	1.05001 (19)	0.0485 (4)	
H14A	0.5629	−0.2265	1.0509	0.073*	
H14B	0.7023	−0.1440	1.1450	0.073*	
H14C	0.7431	−0.2282	1.0210	0.073*	
C15	0.6072 (3)	−0.3610 (2)	0.7190 (3)	0.0648 (5)	
H15A	0.7103	−0.3776	0.6906	0.097*	
H15B	0.5426	−0.4367	0.7113	0.097*	
H15C	0.6289	−0.3319	0.8172	0.097*	
C1	0.46449 (18)	−0.06799 (17)	0.82978 (17)	0.0403 (3)	
H1	0.4716	−0.0145	0.7470	0.048*	
C2	0.3450 (2)	−0.1765 (2)	0.7955 (2)	0.0521 (4)	
H2A	0.2344	−0.1481	0.8005	0.063*	
H2B	0.3768	−0.2417	0.8675	0.063*	
C3	0.3418 (3)	−0.2314 (3)	0.6452 (3)	0.0644 (6)	
H3A	0.2744	−0.3068	0.6327	0.077*	
H3B	0.2905	−0.1712	0.5724	0.077*	
C4	0.5134 (3)	−0.2628 (2)	0.6226 (2)	0.0537 (4)	
C5	0.5937 (2)	−0.1649 (2)	0.55053 (18)	0.0501 (4)	
H5	0.5264	−0.0892	0.5253	0.060*	
C6	0.7768 (2)	−0.14210 (17)	0.57840 (17)	0.0447 (4)	
H6	0.8377	−0.2171	0.6195	0.054*	
C7	0.82874 (19)	−0.02749 (15)	0.67649 (15)	0.0366 (3)	
H7	0.7357	0.0318	0.6601	0.044*	
C8	0.87578 (18)	−0.05478 (18)	0.83958 (15)	0.0393 (3)	
H8A	0.8723	−0.1449	0.8535	0.047*	
H8B	0.9894	−0.0279	0.8751	0.047*	
C9	0.76603 (17)	0.00787 (16)	0.93218 (15)	0.0352 (3)	
H9	0.8333	0.0204	1.0295	0.042*	
C10	0.61532 (17)	−0.06993 (15)	0.94522 (15)	0.0356 (3)	
C11	0.9645 (2)	0.02787 (18)	0.61024 (19)	0.0464 (4)	
C12	0.9353 (3)	−0.0178 (2)	0.4586 (2)	0.0559 (5)	
C13	1.0911 (3)	0.1006 (3)	0.6654 (3)	0.0665 (6)	
H13A	1.1656	0.1250	0.6091	0.080*	
H13B	1.1060	0.1276	0.7607	0.080*	
C16	0.8244 (2)	0.22159 (18)	0.9006 (2)	0.0460 (4)	
C17	0.7628 (3)	0.3407 (2)	0.8271 (2)	0.0597 (5)	
H17A	0.7451	0.4011	0.8976	0.090*	
H17B	0.6600	0.3254	0.7600	0.090*	
H17C	0.8432	0.3726	0.7760	0.090*	
O1	0.5260 (3)	−0.2753 (2)	0.47208 (17)	0.0731 (5)	
O2	0.47027 (14)	−0.00130 (14)	0.96360 (14)	0.0473 (3)	
O3	0.8194 (2)	−0.10899 (18)	0.44012 (14)	0.0622 (4)	
O4	0.9965 (3)	0.0176 (3)	0.36135 (19)	0.0840 (6)	
O5	0.71087 (14)	0.12924 (11)	0.87370 (13)	0.0387 (2)	
O6	0.96035 (19)	0.20593 (19)	0.9760 (2)	0.0711 (5)	

### Atomic displacement parameters (Å^2^)

**Table:**

	*U*^11^	*U*^22^	*U*^33^	*U*^12^	*U*^13^	*U*^23^
C14	0.0535 (9)	0.0498 (10)	0.0430 (8)	0.0018 (8)	0.0119 (7)	0.0129 (7)
C15	0.0920 (16)	0.0371 (9)	0.0689 (13)	0.0029 (10)	0.0251 (12)	0.0000 (9)
C1	0.0353 (6)	0.0393 (7)	0.0477 (7)	0.0015 (6)	0.0119 (5)	0.0030 (6)
C2	0.0378 (7)	0.0526 (10)	0.0660 (11)	−0.0065 (7)	0.0109 (7)	0.0037 (9)
C3	0.0513 (10)	0.0673 (14)	0.0683 (12)	−0.0167 (10)	−0.0022 (9)	−0.0061 (11)
C4	0.0654 (11)	0.0462 (10)	0.0474 (9)	−0.0088 (8)	0.0072 (8)	−0.0093 (7)
C5	0.0593 (9)	0.0500 (10)	0.0377 (7)	−0.0004 (8)	0.0028 (7)	0.0003 (7)
C6	0.0588 (9)	0.0410 (8)	0.0367 (7)	0.0087 (7)	0.0156 (6)	0.0014 (6)
C7	0.0414 (6)	0.0376 (7)	0.0331 (6)	0.0092 (5)	0.0134 (5)	0.0039 (5)
C8	0.0379 (6)	0.0472 (8)	0.0340 (6)	0.0098 (6)	0.0105 (5)	0.0069 (6)
C9	0.0367 (6)	0.0383 (7)	0.0307 (5)	−0.0009 (5)	0.0077 (4)	−0.0002 (5)
C10	0.0374 (6)	0.0353 (7)	0.0366 (6)	0.0030 (5)	0.0135 (5)	0.0008 (5)
C11	0.0546 (8)	0.0436 (8)	0.0470 (8)	0.0113 (7)	0.0244 (7)	0.0111 (7)
C12	0.0688 (11)	0.0586 (12)	0.0482 (9)	0.0182 (10)	0.0303 (8)	0.0098 (8)
C13	0.0757 (14)	0.0592 (13)	0.0730 (13)	−0.0089 (11)	0.0347 (11)	0.0052 (11)
C16	0.0531 (9)	0.0411 (8)	0.0491 (8)	−0.0111 (7)	0.0231 (7)	−0.0132 (7)
C17	0.0799 (13)	0.0397 (9)	0.0674 (12)	−0.0121 (10)	0.0336 (10)	−0.0033 (9)
O1	0.0935 (12)	0.0769 (12)	0.0452 (8)	−0.0166 (11)	0.0059 (7)	−0.0190 (8)
O2	0.0473 (6)	0.0429 (6)	0.0586 (7)	0.0051 (5)	0.0267 (5)	−0.0034 (6)
O3	0.0873 (10)	0.0679 (10)	0.0372 (6)	0.0057 (9)	0.0268 (6)	−0.0042 (6)
O4	0.1043 (14)	0.1021 (15)	0.0613 (9)	0.0130 (13)	0.0531 (10)	0.0150 (11)
O5	0.0410 (5)	0.0329 (5)	0.0432 (5)	−0.0035 (4)	0.0110 (4)	−0.0027 (4)
O6	0.0544 (8)	0.0626 (10)	0.0909 (13)	−0.0184 (8)	0.0025 (8)	−0.0182 (9)

### Geometric parameters (Å, °)

**Table:**

C14—C10	1.504 (2)	C6—C7	1.539 (2)
C14—H14A	0.9600	C6—H6	0.9800
C14—H14B	0.9600	C7—C11	1.511 (2)
C14—H14C	0.9600	C7—C8	1.5386 (19)
C15—C4	1.493 (3)	C7—H7	0.9800
C15—H15A	0.9600	C8—C9	1.537 (2)
C15—H15B	0.9600	C8—H8A	0.9700
C15—H15C	0.9600	C8—H8B	0.9700
C1—O2	1.444 (2)	C9—O5	1.442 (2)
C1—C10	1.474 (2)	C9—C10	1.519 (2)
C1—C2	1.508 (3)	C9—H9	0.9800
C1—H1	0.9800	C10—O2	1.4419 (18)
C2—C3	1.534 (3)	C11—C13	1.316 (3)
C2—H2A	0.9700	C11—C12	1.486 (3)
C2—H2B	0.9700	C12—O4	1.199 (2)
C3—C4	1.511 (3)	C12—O3	1.348 (3)
C3—H3A	0.9700	C13—H13A	0.9300
C3—H3B	0.9700	C13—H13B	0.9300
C4—O1	1.456 (3)	C16—O6	1.207 (3)
C4—C5	1.476 (3)	C16—O5	1.345 (2)
C5—O1	1.439 (3)	C16—C17	1.484 (3)
C5—C6	1.496 (3)	C17—H17A	0.9600
C5—H5	0.9800	C17—H17B	0.9600
C6—O3	1.467 (2)	C17—H17C	0.9600
			
C10—C14—H14A	109.5	C7—C6—H6	110.6
C10—C14—H14B	109.5	C11—C7—C8	115.88 (14)
H14A—C14—H14B	109.5	C11—C7—C6	101.30 (13)
C10—C14—H14C	109.5	C8—C7—C6	115.81 (14)
H14A—C14—H14C	109.5	C11—C7—H7	107.8
H14B—C14—H14C	109.5	C8—C7—H7	107.8
C4—C15—H15A	109.5	C6—C7—H7	107.8
C4—C15—H15B	109.5	C9—C8—C7	115.78 (12)
H15A—C15—H15B	109.5	C9—C8—H8A	108.3
C4—C15—H15C	109.5	C7—C8—H8A	108.3
H15A—C15—H15C	109.5	C9—C8—H8B	108.3
H15B—C15—H15C	109.5	C7—C8—H8B	108.3
O2—C1—C10	59.23 (10)	H8A—C8—H8B	107.4
O2—C1—C2	117.75 (14)	O5—C9—C10	108.82 (11)
C10—C1—C2	124.07 (16)	O5—C9—C8	110.26 (12)
O2—C1—H1	114.7	C10—C9—C8	113.44 (13)
C10—C1—H1	114.7	O5—C9—H9	108.1
C2—C1—H1	114.7	C10—C9—H9	108.1
C1—C2—C3	112.05 (17)	C8—C9—H9	108.1
C1—C2—H2A	109.2	O2—C10—C1	59.36 (10)
C3—C2—H2A	109.2	O2—C10—C14	113.50 (13)
C1—C2—H2B	109.2	C1—C10—C14	123.48 (15)
C3—C2—H2B	109.2	O2—C10—C9	116.46 (13)
H2A—C2—H2B	107.9	C1—C10—C9	120.62 (13)
C4—C3—C2	112.34 (16)	C14—C10—C9	112.01 (13)
C4—C3—H3A	109.1	C13—C11—C12	122.04 (18)
C2—C3—H3A	109.1	C13—C11—C7	131.20 (19)
C4—C3—H3B	109.1	C12—C11—C7	106.75 (17)
C2—C3—H3B	109.1	O4—C12—O3	121.8 (2)
H3A—C3—H3B	107.9	O4—C12—C11	129.0 (3)
O1—C4—C5	58.81 (14)	O3—C12—C11	109.18 (15)
O1—C4—C15	113.58 (19)	C11—C13—H13A	120.0
C5—C4—C15	123.53 (19)	C11—C13—H13B	120.0
O1—C4—C3	114.76 (18)	H13A—C13—H13B	120.0
C5—C4—C3	115.6 (2)	O6—C16—O5	122.27 (19)
C15—C4—C3	116.7 (2)	O6—C16—C17	125.43 (18)
O1—C5—C4	59.90 (14)	O5—C16—C17	112.30 (16)
O1—C5—C6	119.44 (19)	C16—C17—H17A	109.5
C4—C5—C6	124.49 (17)	C16—C17—H17B	109.5
O1—C5—H5	114.1	H17A—C17—H17B	109.5
C4—C5—H5	114.1	C16—C17—H17C	109.5
C6—C5—H5	114.1	H17A—C17—H17C	109.5
O3—C6—C5	107.57 (14)	H17B—C17—H17C	109.5
O3—C6—C7	105.02 (14)	C5—O1—C4	61.28 (13)
C5—C6—C7	112.25 (14)	C10—O2—C1	61.41 (10)
O3—C6—H6	110.6	C12—O3—C6	110.63 (14)
C5—C6—H6	110.6	C16—O5—C9	115.58 (13)
